# A Financial Case for a Medical-Legal Partnership: Reducing Lengths of Stay for Inpatient Care

**DOI:** 10.1017/jme.2023.148

**Published:** 2023

**Authors:** Barak D. Richman, Breanna Barrett, Riya Mohan, Devdutta Sangvai

**Affiliations:** 1: DUKE UNIVERSITY SCHOOL OF LAW, DURHAM, NC, USA; 2: CLINICAL EXCELLENCE RESEARCH CENTER, STANFORD UNIVERSITY SCHOOL OF MEDICINE, PALO ALTO, CA, USA; 3: GEORGE WASHINGTON SCHOOL OF LAW, WASHINGTON, DC, USA; 4: DUKE UNIVERSITY, DURHAM, NC, USA; 5: DUKE UNIVERSITY HEALTH SYSTEM, DURHAM, NC, USA.

**Keywords:** Medical Legal Partnership, Inpatient Length of Stay, Social and Legal Determinants of Health

## Abstract

While Medical-Legal Partnerships (MLPs) have improved the health and well-being of the people they serve, most healthcare institutions will only invest in an MLP if they are convinced that doing so will improve its balance sheet. This article offers a detailed estimation of the cost savings that an MLP targeted toward the most acute legal needs would accrue to an academic medical center (AMC) in North Carolina.

Medical-legal partnerships (MLPs) have been shown to improve patient health in ways that reach beyond what medical care alone can do. By providing patients with legal services that, among other benefits, improve patient housing, mobility, employment prospects, and other social drivers of health, MLPs have an established record that should convince most healthcare systems to include certain legal services in their core service offerings.^1^ Nonetheless, many health systems require a financial justification for MLPs. In other words, convincing a health system to invest in an MLP often requires more than showing that these services will meaningfully benefit their patients. In a setting of constrained resources and competing demands, it requires supplying evidence that an MLP will enable the health system to see financial upside that exceeds the expenses of an MLP.

We examined qualitative and quantitative data from an academic medical center (AMC) with an eye to examining whether an MLP might reduce overall cost burdens. We identified a particular metric — the inpatient length of stay (LOS) — as an indicator of how an MLP can improve a health system’s finances. A patient’s LOS reflects a quantifiable cost associated with the length of a patient’s care, and reductions in the days of care offers a financial metric for savings that accrue from a prompt discharge. If MLPs can expedite the transition of inpatients to either step-down facilities or home settings, then — in addition to the obvious psychological and physiological benefits that would accrue to the patient — the health system would both avoid unnecessary medical expenses and increase access to inpatient services for new patients.

This article examines the financial case for MLPs in three parts. Part One explains the methodology behind our study: We consulted with national leaders who have constructed successful MLPs to estimate the costs of building a new MLP at an AMC and reviewed the literature to determine how MLPs might improve patient care. We then interviewed social workers at an AMC to understand where patients exhibit needs for legal services and how supplying those legal services might reduce costs. This investigation revealed how providing legal services can fruitfully reduce patients’ inpatient stays and how our metric — length of stay (LOS) — can serve as an accounting measure to make a financial case to AMC leaders. That examination also suggested that the legal services that could lead to an immediate reduction in average LOS are those that clarify patients’ guardianship status. In Part Two, we explore state guardianship procedures, examine delays that typify the AMC’s experience in pursuing guardianship proceedings, and estimate how providing legal services to patients needing changed guardianship status will impact average LOS and affect AMC costs. However, precisely because guardianship is an extreme instrument in that it strips certain capabilities of legal self-determination from patients, we close in Part Three with some cautionary remarks about using MLP resources to facilitate guardianship proceedings, especially if designed to expedite patients’ discharges from inpatient care.

The core of our analysis is a financial cost-benefit assessment that estimates whether an AMC will see overall savings by investing in an MLP. In this section, we first document how we estimate the costs of an MLP, and then describe our data, metrics, and calculations that underlie our estimate of MLP-generated savings.

## I. Methods: Calculating the Costs and Estimating the Benefits of an MLP

The core of our analysis is a financial cost-benefit assessment that estimates whether an AMC will see overall savings by investing in an MLP. In this section, we first document how we estimate the costs of an MLP, and then describe our data, metrics, and calculations that underlie our estimate of MLP-generated savings.

### A. Calculating the Costs of an MLP

To estimate total annual costs for an MLP at an AMC, we consulted national experts who have built, managed, and documented the effectiveness of MLPs at other AMCs. In particular, we inquired into the budgetary requirements to sustain a variety of MLPs and derived common features that would readily apply to our home AMC. We concluded that the costs of sustaining an MLP at an AMC would require a personnel team of six professionals, funded at a variety of levels. Although our budget would support personnel fringe benefits, it would not require capital or overhead expenditures. For the purpose of estimating the total operating costs, we assumed that the MLP would be at capacity for the entire year.

We adopted the personnel model to average wages in the southeast region of the United States. The fringe benefit rate was a sample non-federal, projected rate for FY 2024-25,^2^ and specific salary averages were derived from a salary report database with job-specific wages adjusted to various US cities.^3^ The total amount estimated to be required for staffing an MLP can be found in Table 1.

**Table 1:** Annual Costs of a Medical-Legal Partnership in the Southeastern United States, *Annual Budget in* USD

Program Development Research & Writing, Substantive Planning Administrative Costs	**Salary**^4^ ***(12 Months)***	Fringe Benefits (%)	Percent Effort (%)	Blended Salary for Budget	**Salary & Fringe Benefits Requested** ^5^
Faculty Director	$165,000	25.5%	5%	$168,300	$16,998
Attorney	$159,890	25.5%	100%	$163,088	$203,860
Hospital Social Worker	$66,832	25.5%	20%	$68,168	$17,110
Medical Administrator	$54,234	25.5%	20%	$55,319	$13,885
Program Evaluator (1-2 employees)	$59,635	25.5%	20%	$60,828	$15,268
Paralegal	59,264	25.5%	100%	$60,449	$75,864
Law Students & Junior Lawyers	$0	0%	100%	$0	$0
**Total Amount:**					**$342,985**

**Table 2:** Calculated Annual Savings from MLP Based on Reduced Length of Stays

LOS Savings/Reduced day	MLP Beneficiaries/Year	Average Reduced LOS/Person	Total
$825	75	20	**$1,237,500**

### B. Calculating the Savings from an MLP: Socio-Legal Needs and Extended Length of Stay (LOS)

We began our investigation into the benefits of an MLP by interviewing healthcare team members familiar with extended LOS caused by nonmedical conditions. Our first rounds of interviews were with care coordinators and social workers who identified various legal needs that, in their judgment, most significantly affected patient care health outcomes and their courses of care. These discussions identified five socio-legal determinants of health that arose most frequently in patient care: housing, guardianship, disabilities, immigration status, and expungement of criminal records.

We then investigated how these five separate legal needs affected patient courses of care, inquiring with care managers in interviews and examining health system accounting data. We learned that when these legal needs go unmet, one severe consequence is a delay in discharge: Patients find themselves unable to transition out of inpatient care to subsequent step-down facilities, such as those offering skilled nursing, behavioral health, or in-home therapy. Some patients cannot gain admission to a rehabilitation facility because they have not been approved for public benefits (and thereby cannot pay for the facility); others live in substandard or insecure housing and thus would not recover if sent home to their primary residence; and others have disabilities but can only manage self-care if they qualify for and receive disability benefits. When these delays occur because of lack of legal services, not only do hospitals bear additional costs, but patients unnecessarily suffer since step-down facilities or in-home care often accelerate the healing process.^6^

These inquiries generated perhaps the most significant finding in our examination: one consequence of unmet legal needs is an extended inpatient length of stay (LOS). We therefore identified extended length of stay as a standard financial metric that measures the financial costs to an AMC of failing to meet their patients’ legal needs.

#### background on length of stay

Length of stay (LOS) reflects the period during which a patient remains in an inpatient facility, from admission to discharge. Commonly measured in days to the first decimal place (e.g. 3.6 days), LOS can be used in operational and financial functions for the facility. For example, LOS can reflect the hospital stay’s overall efficiency and predict volumes for future forecasting.^7^ The observed LOS (OLOS) is the actual number of days a patient stayed in the hospital, compared to the expected LOS (ELOS), which is a predetermined value largely based on the clinical condition(s) for which the patient is admitted and the overall complexity of the case.^8^ When the OLOS exceeds ELOS, LOS is considered extended.

Most inpatient admissions for Medicare beneficiaries are paid in accordance with the Medicare Severity-Diagnostic Related Group (MS-DRG) model. Under the DRG framework, a facility is paid a fixed amount for services rendered to a patient with a given diagnosis, so the facility is not financially incentivized to provide unnecessary diagnostics, services, or interventions. Included in this are days in the hospital. If a patient is in the hospital for more days than expected without a clinical rationale or medical reason, the hospital does not get paid for those additional days.

Hospitals frequently employ financial accounting that tracks expenses at a patient level, which allows them to calculate a cost per case (CPC). Dividing the CPC by the length of stay yields the expense per day (EPD) of an occupied hospital bed for a particular patient. Hospitals then calculate the expenses associated with an extended LOS by multiplying the EPD by the difference between OLOS and ELOS. Consulting hospital accounting data, we estimate that an additional day of inpatient care costs the AMC $825. So, if a patient is admitted for a condition that has an ELOS of 5 days but is discharged after 8 days, and the hospital’s EPD is $825/day, the cost of the extended LOS is (8 days - 5 days) * $825/day = $2,475. This $2,475 represents an avoidable expense. For every day that the OLOS exceeds the ELOS, this expense increases at a linear rate.

#### los and guardianship

We consulted health system data to identify factors associated with extended inpatient LOS and compared them to our top five social-legal factors. Among these five, “guardianship” was the primary factor, and this was confirmed with subsequent interviews with case managers. Patients coded as experiencing extended LOS due to “guardianship” are patients deemed by medical personnel to be cognitively impaired such that they could not agree to a discharge statement, approve a transfer to another facility, secure public benefits that could pay for a step-down facility, or care for themselves at home. Most of these patients cannot transition to more appropriate facilities until the state assigns a guardian who can manage the patient’s legal and personal affairs. After identifying our financial metric of interest (LOS) and the legal need of interest (guardianship), the remainder of our analysis was defined: we then examined how providing patients with legal services might facilitate the guardianship process and reduce these patients’ inpatient LOS.

## II. Guardianship and Inpatient Costs

In this section, we examine whether an MLP would responsibly reduce the prolonged LOS for patients who are not cognitively competent to approve their own discharge. We additionally estimate whether an MLP would reduce extended LOS such that hospital savings would outweigh the costs of an MLP. This section describes the patient population that requires guardianship and the costs that accrue to an AMC when those needs are unmet. It then estimates the savings an MLP would generate by providing legal services to resolve guardianship challenges.

### A. The Cost of Increased LOS for Patients in Need of Guardians

Patients suffering from cognitive impairments that prevent them from approving their own discharges tend to be older patients who lack close family advocates or who have complex social needs or backgrounds that are further impeded by guardianship issues. Many have complex medical histories, legal issues, or extenuating circumstances that limit their ability to access federal benefits. Thus, transferring these patients to more appropriate and rehabilitative settings — thereby improving patient wellbeing and reducing hospital costs — requires resolving guardianship status and often other legal barriers that impede discharge. The key legal barriers related to navigating guardianship procedures include identifying appropriate guardians for the patient, addressing instances of abuse or neglect with current guardians, and finding a court-appointed guardian in especially complicated circumstances.

#### the amc experience

The care delivery process is fairly complex, especially since most cases with guardianship issues concern older patients who have complicated medical and social histories. Most often, the patient is flagged by the physician treating them, social work team, or the patient themself. Once the AMC’s social work or complex care unit is involved and a medical affidavit proving the patient’s impairment is obtained, the next of kin or the patient’s appointed power of attorney is identified.

Late discharges become inevitable when the AMC cannot identify a next of kin or designated power of attorney, since the process triggered by the patient’s lack of guardian subsequently requires the involvement of a court to identify a suitable guardian. From the data collected by the AMC, there are roughly 75-100 patients each year that have extended LOS directly due to the patient’s inability to make competent decisions and their lack of a guardian who can function as an alternative medical decision-maker.

### B. LOS Savings from MLP Guardianship Services

A guardian can serve as a substitute decision-maker for individuals who lack competency. When a court determines that an adult is incapable of managing their own affairs, such as making significant decisions regarding their lives, family, or property, a guardian is appointed to make legal and medical decisions on their behalf. In North Carolina, guardians are appointed by the clerk of the superior court, with powers and duties varying by type of guardianship.^9^ The powers and duties of Guardians of the Estate are outlined in the law and differ from the powers of general guardians who have the powers of both a Guardian of the Person and a Guardian of the Estate.^10^

However, the process for conferring guardianship has many limitations and legal requirements, which result in delays for patients who require guardians in order to be discharged. In North Carolina, a guardian can be appointed only after a ward and other appropriate parties are served by an officer of the court, and this service can take place no fewer than 10 days and no more than 30 days after the filing of a petition with the clerk of court.^11^ Because of delays, largely attributable to the shortage of legal services and lack of attention from lawyers, most guardianship cases take more than 30 days rather than the 10-day minimum. We estimate that an MLP with a dedicated attorney handling guardianship cases would accelerate this process, such that legal proceedings that typically take more than 30 days are cut down to only 10 days. We therefore assume that, for patients whose discharges are delayed due to guardianship delays, an MLP will expedite patients’ transfer to a more appropriate facility by 20 days.

With this information, we can estimate the full savings that an MLP would generate for patients with guardianship needs alone. The AMC would save $825 for each reduced day of an inpatient stay, and approximately 75 patients a year have extended inpatient stays because of guardianship issues; since these extended stays often exceed 30 days, we estimate an MLP would reduce LOS by 20 days. This would lead to an overall expense reduction of ($825/day) x (75 patients) x (20 days/patient) = $1,237,500 at a cost of $342,985. Because we use conservative estimates for the cost of an additional LOS, the number of patients affected annually, and the days delayed by guardianship proceedings, we believe that the actual savings would be much higher.

## III. Conclusion: A Word of Caution on Guardianship

Our financial calculation suggests significant savings would accrue to an AMC — in addition to the health benefits that would accrue to a patient — if an MLP were to expedite the process of assigning guardians to cognitively impaired patients that cannot authorize their own hospital discharge. We conservatively estimate that the AMC in our study would enjoy over $1.2 million in savings from guardianship cases alone, over three times the annual cost of an MLP, and we expect that AMCs and other hospital systems with similar cost structures would accrue comparable savings.

Nonetheless, we must include serious reservations about expanded access to the guardianship process.^12^ Experts in guardianship law have warned us that the guardianship process in North Carolina suffers from excessive, not limited access, and that hospitals have too readily and easily stripped their patients of their legal rights. Indeed, guardianship is an extreme measure that deprives patients of their legal autonomy. In fact, some North Carolina hospitals use social workers and case managers to lead the court processes, and guardianship proceedings advance without a judge or close lawyerly scrutiny. Many have argued that the process offers inadequate protection for impaired individuals, and experts have advocated for reforming guardianship ad litem procedures to make guardianship more difficult to procure.

Moreover, a severe conflict of interest arises if a health system were to employ MLP personnel whose role was then to manage guardianship proceedings to expedite their patients’ discharge. If hospital personnel try to transfer patients to other facilities too quickly, the health system is at risk of not fulfilling its duty to treat and care for its patients. Precisely because guardianship is a severe instrument, an MLP that represents the interests of patients would need to be independent of the AMC which would accrue financial benefits from prompt discharges.

We take these dynamics seriously, and we close with suggestions for how MLPs might navigate these hazards effectively. First, we believe that an MLP, with trained attorneys, could bring more professionalism and care to a process that regularly lacks attorney oversight.^13^ We encourage any MLP that manages a guardianship proceeding for a patient-client to also ensure that a trained attorney would serve as the patient’s guardian ad litem.^14^ It is possible that this arrangement would not only advance both the hospital’s mission and the patient’s welfare but would also establish greater integrity for the guardianship process. Second, we recommend that MLP personnel are employed by a third-party organization, such as the local Legal Aid institution, to ensure that MLP personnel are subject to avenues of accountability separate from AMC leadership. This would ensure that any MLP attorney would represent the interests of their client-patients alone, and that any financial arrangement between the AMC and the MLP would be managed by disinterested parties. Finally, implementing an MLP would require further evaluation and follow-up with patients after discharge. Our financial model only considers previously collected data regarding patient care and experiences. In order to fully understand the impact and benefit (or possible harms) an MLP could create for the AMC, a well-structured data collection and evaluation system should broadly monitor its financial and health consequences.

## Note

The authors have no conflicts to disclose.
